# Optimized Surgical Outcomes in Living Donor Nephrectomy: A Single-Center Experience with 250 Cases Using a Novel Technique

**DOI:** 10.5152/tud.2025.25030

**Published:** 2025-06-04

**Authors:** Amil Huseynov

**Affiliations:** 1 Department of Transplantation, Medicana Hospital, İstanbul Türkiye; 2 Beykoz University Faculty of Medicine, İstanbul,Türkiye

**Keywords:** Laparoscopic donor nephrectomy, living kidney donation, minimally invasive surgery, modified surgical technique, postoperative outcomes

## Abstract

**Objective:**

Laparoscopic donor nephrectomy has become a standard of care for living kidney donors, providing reduced morbidity, quicker recovery, and enhanced patient satisfaction compared with open techniques. This study evaluates a modified laparoscopic donor nephrectomy technique designed to minimize colon mobilization while optimizing visualization, thereby improving donor outcomes.

**Methods:**

A cross-sectional study of 250 consecutive living donor nephrectomies performed by a single surgeon between March 2022 and March 2024 was conducted. All donors underwent preoperative imaging (3D computed tomography and computed tomographic angiography). The modified approach avoided splenic flexure dissection and introduced early ligation of the adrenal vein. We analyzed intraoperative parameters (operative time, estimated blood loss, complications) and postoperative measures (hospital stay, pain scores, complication rates, satisfaction) were analyzed.

**Results:**

The mean operative time was 72.8 ± 16.2 minutes, with an estimated blood loss of 100 ± 40 mL. No conversions to open surgery or intraoperative transfusions were required. The mean length of hospital stay was 2.0 ± 1.0 days. Postoperative pain (mean Visual Analog Scale [VAS] 2.5 ± 1.8) was low, and major vascular injury occurred in 0.8% of cases. Wound infection and incisional hernia rates were each 0.8%. Patient satisfaction was high (Patient Satisfaction Questionnaire [PSQ] 3.3 ± 1.4), indicating favorable perceptions of comfort and outcomes.

**Conclusion:**

This modified laparoscopic donor nephrectomy technique is safe, effective, and associated with enhanced patient comfort and reduced complications. The findings underscore its potential to improve donor experiences, potentially expanding the living donor pool. Further investigations should confirm these outcomes over a longer follow-up period.

**Main Points**

- Refined Surgical Approach: The modified laparoscopic technique reduces the need for extensive mobilization, particularly avoiding splenic flexure dissection, which shortens operative times and lowers the risk of complications.
- Improved Donor Comfort: Targeted dissection and limited mobilization contributed to low pain scores (Visual Analog Scale [VAS] 2.5 ± 1.8) and short hospital stays (2.0 ± 1.0 days), indicating enhanced postoperative comfort and faster recovery for living donors.
- Enhanced Safety Profile: Clear visualization of the adrenal vein through initial adrenal dissection, combined with minimized handling of surrounding structures, resulted in low complication rates (intraoperative 0.8%, postoperative 2.8%).
- High Patient Satisfaction: The high Patient Satisfaction Questionnaire score (3.3 ± 1.4) reflects donors’ positive experiences, suggesting that this technique can help alleviate concerns about pain and recovery, potentially expanding the living donor pool.

## Introduction

Laparoscopic donor nephrectomy has become a cornerstone of contemporary kidney transplantation, offering significant reductions in both morbidity and mortality since its initial introduction.^1^ These advances have contributed to a global rise in living kidney donation, driven largely by the adoption of minimally invasive surgical approaches.^2^ Numerous studies comparing laparoscopic and open donor nephrectomy have demonstrated superior short- and long-term outcomes for the laparoscopic technique.^3^ These benefits include accelerated postoperative recovery, reduced hospital stays, and diminished postoperative pain, collectively enhancing donor safety and satisfaction.^4^ Additionally, the laparoscopic approach has been associated with lower rates of complications and infection.^5^^-^^8^

In this case series, a modified laparoscopic donor nephrectomy technique was presented that was designed to further optimize donor comfort and minimize postoperative complications. Short-term surgical outcomes were retrospectively analyzed from a single surgeon’s experience, with a focus on key technical refinements and their clinical impact. By sharing these findings, the aim is to encourage broader adoption of laparoscopic donor nephrectomy, thereby improving donor experiences and surgical outcomes in the field of kidney transplantation.

## Material and Methods

This cross-sectional study was conducted at Medicana Hospital between March 2022 and March 2024. The study protocol was in accordance with the Declaration of Helsinki and was approved by the Beykoz University Institutional Review Board (Aproval No.: 2022/21 Date: June 8, 2022). Written informed consent was obtained from all participants prior to inclusion in the study. All donor cases that met the donor criteria were included, without any specific exclusion criteria. Data was collected from medical records, including preoperative evaluations and testing, such as 3D computed tomography and computed tomographic angiography. Pre-, intra-, and post-operative parameters were analyzed, including donor and recipient kidney function, duration of surgery, length of hospital stays, and complication rates.

To assess patient comfort, the Visual Analog Scale (VAS) for postoperative pain, the Hospital Anxiety and Depression Scale (HADS), and the Patient Satisfaction Questionnaire (PSQ) were used.

All cases were operated with the modified technique.

### Surgical Technique

The patient is placed under general anesthesia and positioned in the lateral decubitus position with the side of the donor kidney facing up. The lower extremity is slightly flexed, and the upper extremity is extended and secured, allowing the surgeon easy access to the kidney and associated structures. The surgeon then places 3 trocars in the abdominal wall. The first trocar is typically inserted in the umbilical region, while the other trocars are positioned according to the kidney’s anatomy and the surgeon’s technical preferences. After the trocars are in place, the surgeon insufflates the abdominal cavity with CO_2_ to create a laparoscopic workspace. Following this, the procedure moves to the 3-step section according to the modified technique.

**Figure 1.**
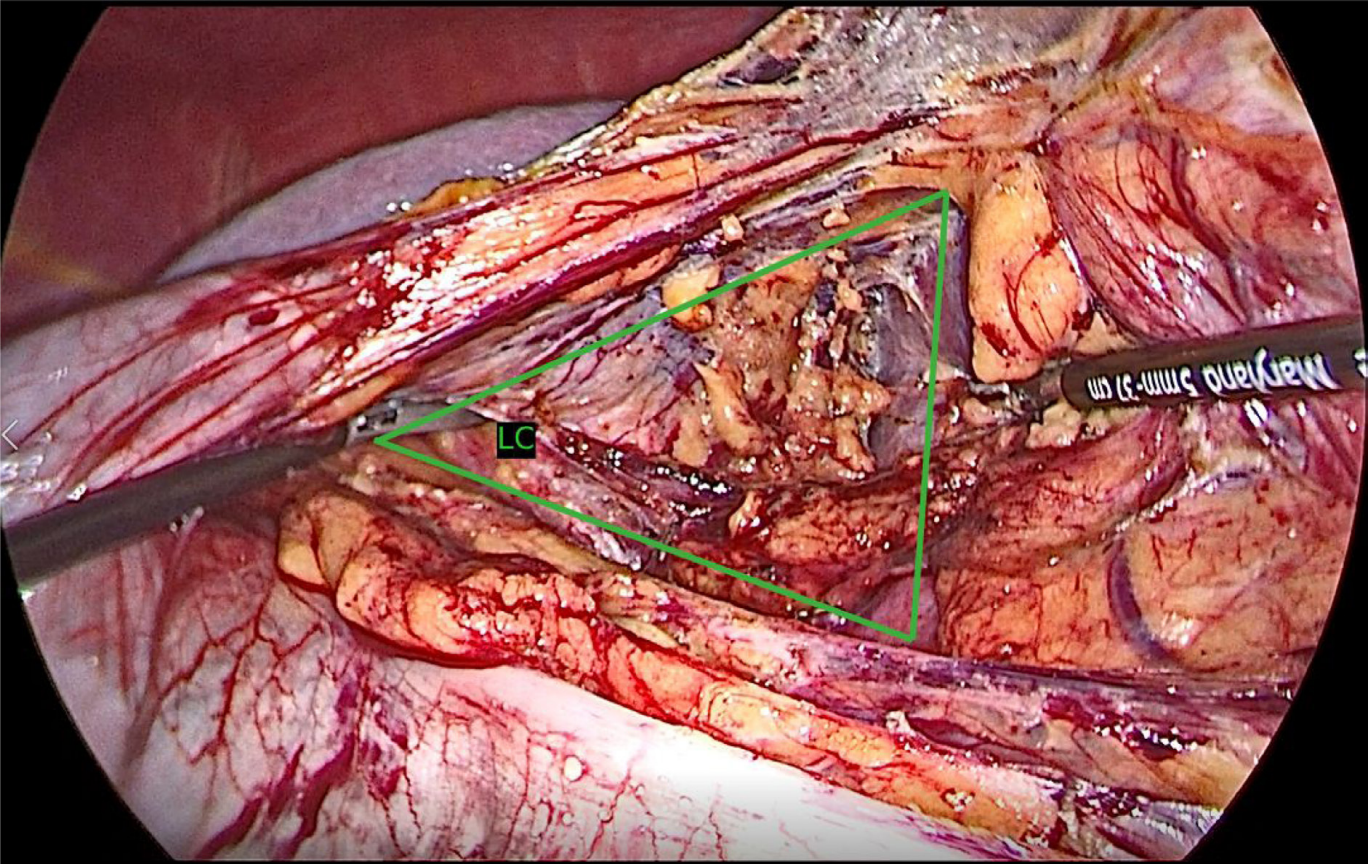
Upper pole dissection is performed without dropping the splenic flexure of the left colon. Toldt’s fascia is dissected, forming a triangular configuration, which allows better visualization and manipulation during the procedure. LC, left colon.

Without dropping the splenic flexure of the left colon, the Toldt’s fascia is dissected, forming a triangular configuration (Figure 1).

**Figure 2.**
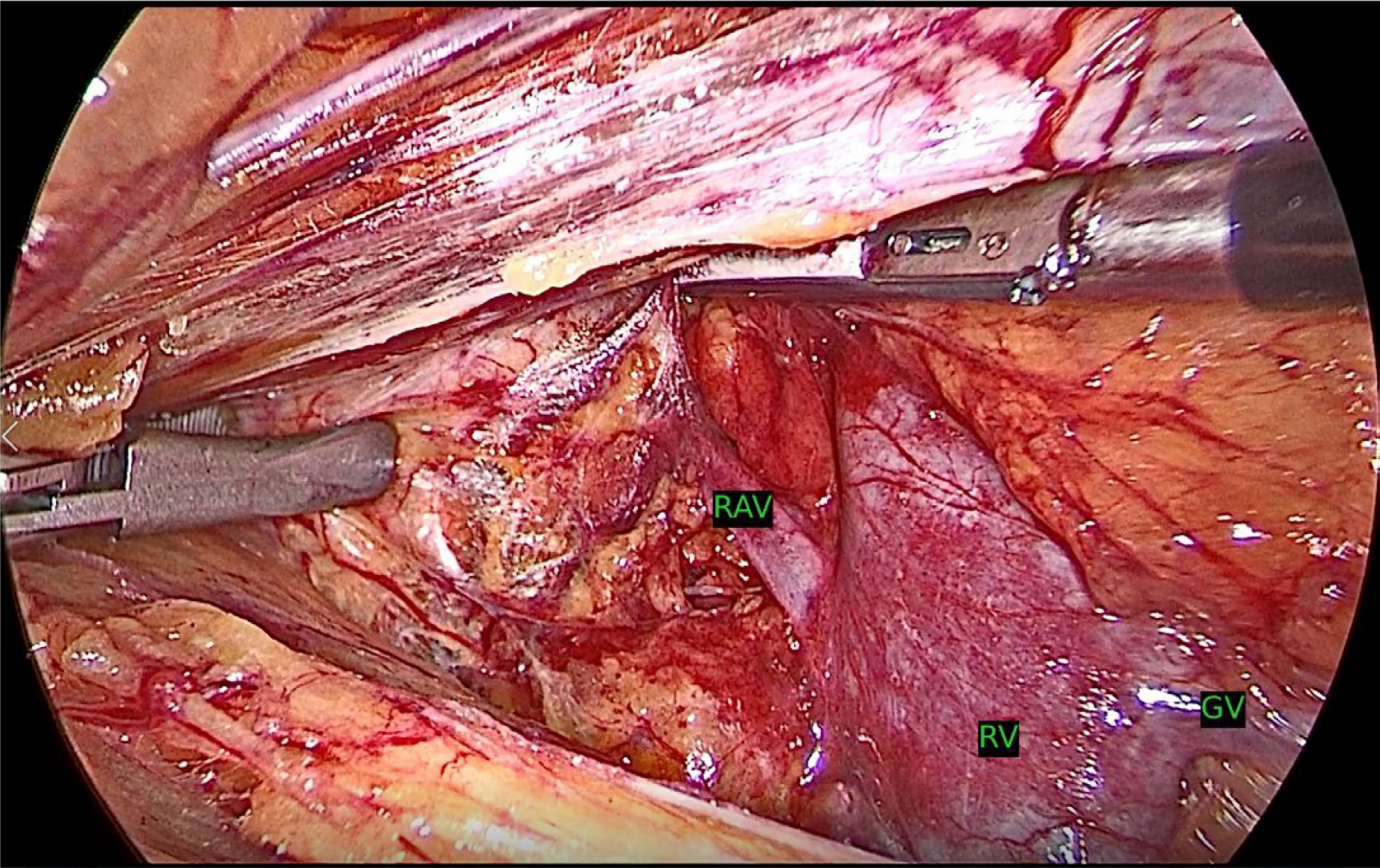
The modified technique involves dissecting and ligating the adrenal vein first, followed by the gonadal vein dissection. This method enhances the visualization of the adrenal vein due to the traction exerted by the gonadal vein located inferior to the renal vein. This approach allows for easier manipulation during dissection in the event of significant vascular trauma. GV, gonadal vein; RAV, adrenal vein; RV, renal vein.

In this modified technique, the upper and lower poles of the kidney are freed without performing hilum dissection. This approach allows for easier manipulation during dissection in case of significant vascular trauma (Figure 2).

**Figure 3.**
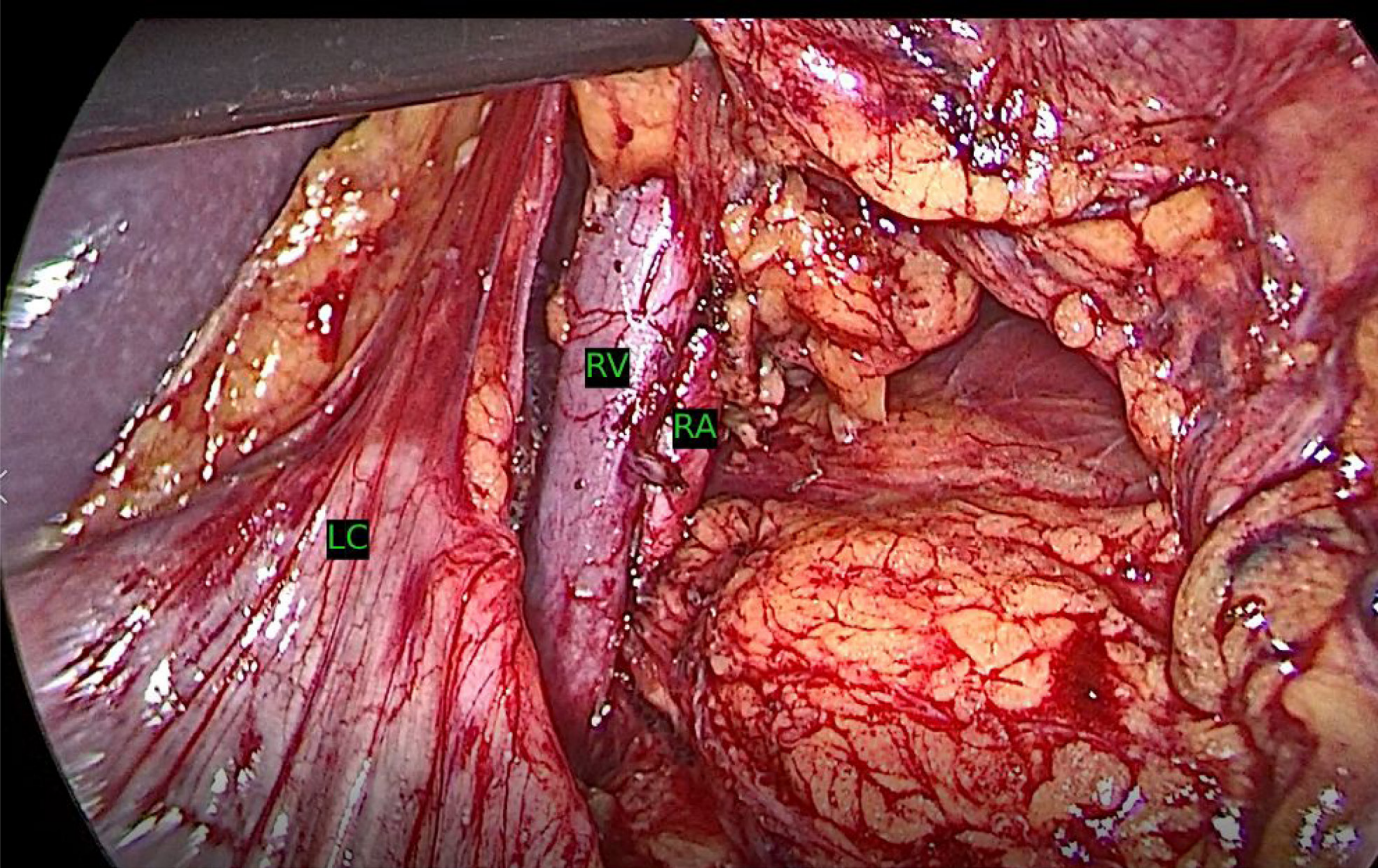
Final state of the dissection before kidney removal, showing the completed dissection and ligation of the adrenal and gonadal veins. The enhanced visualization of the adrenal vein achieved through this technique is evident, facilitating the safe removal of the kidney.

In addition to the standard technique, the first step is to ligate and divide the adrenal vein, followed by the gonadal vein dissection. This approach improves visualization of the adrenal vein due to the traction of the gonadal vein (Figure 3).

From this point, the procedure continues according to the standard technique. Before removing the kidney, the dissection is completed, and the first incision is made. Following this, the ureter is clamped and divided. Subsequently, the renal artery and vein are ligated and divided using Hem-o-lok clips or other appropriate clamps, thus devascularizing the kidney. The ligated and divided kidney is then carefully placed in an endoscopic bag and removed through the trocar opening. An expanded trocar site or a Pfannenstiel incision can be used to extract the kidney. Before closing, pneumoperitoneum is re-established to inspect the dissection area, and a JP drain is placed in the renal fossa. After the kidney is removed, the surgeon evacuates the CO_2_ from the abdominal cavity and closes the trocar incisions. The patient is then monitored and followed up during the postoperative period.

## Results

We performed 250 laparoscopic donor nephrectomies during the study period. The median age of living donors was 34. In 91% of cases, left donor nephrectomy was performed. The rate of kidneys with cyst and stone formation was 7%, and the rate of patients with multiple renal vessels was 21.5% (Table 1).

The mean operative time was 45 ± 16 minutes, and the mean estimated blood loss was 100 ± 40 ml. No cases required a blood transfusion or conversion to open surgery (Table 2). Intraoperative complications occurred in 0.8% of cases, including major vascular injury in 1 case. The mean length of the hospital stay was 2.0  ±  1.0 days. (Table 3).

The mean VAS score was 2.5 ± 1.8, indicating effective pain management. The mean HADS score was 6.5 ± 4.3, suggesting that most patients experienced mild to moderate anxiety or depression levels. Finally, the mean PSQ score was 3.3 ± 1.4, reflecting a high level of patient satisfaction with the care provided (Table 4).

These data collectively demonstrate that patients reported high comfort and satisfaction levels during their hospital stay for the laparoscopic donor nephrectomy procedure.

## Discussion

The modified technique used in the study involved reaching the hilum without splenic flexure dissection, freeing the upper and lower poles without hilum dissection, and allowing for easy manipulation during dissection in the event of major vascular trauma. This approach not only resulted in a shorter and safer operative time but also required less mobilization of the left colon. This led to increased postoperative patient comfort, as evidenced by scores such as VAS for postoperative pain, HADS, and PSQ. Additionally, the limited mobilization of the left colon was significantly effective in reducing complications such as ileus and prolonged hospital stays.

This technique not only offers advantages over open donor nephrectomy, as demonstrated by improved short- and long-term outcomes, but it also provides superior results compared to other laparoscopic nephrectomy techniques.^1^,^9^^-^^11^ Additionally, it was found that suspending the descending colon without clipping the gonadal vein during adrenal dissection provided clear visualization of the adrenal vein, which could be useful for other surgeons using a similar approach.^12^ The mean length of hospital stay for the patients was notably better than previously reported data, reflecting the effectiveness of the modified technique.^13^,^14^ The shorter recovery time and improved overall comfort of donors are important factors in expanding the donor pool, as donor comfort and social life are crucial considerations for potential donors.^15^^,^^16^ As reported in previous studies,^17^,^18^ better visualization and less invasive techniques often result in improved patient comfort and satisfaction.

However, there are several limitations to the study. The follow-up period was short, so longer-term outcomes, such as patient-reported quality of life measures and the durability of surgical outcomes, should be explored in future research.^19^ Despite these limitations, the study has several strengths that should be highlighted. Being a single-center study allowed for consistency in surgical technique and postoperative care, offering a controlled environment to evaluate the effectiveness of the modified approach. Future comparative studies involving different surgical techniques and surgeons can further validate and expand upon the findings, providing broader insights into the safety and efficacy of various approaches.^20^

This study demonstrates that a single surgeon can safely and effectively perform laparoscopic donor nephrectomy using the modified technique. By limiting extensive mobilization and emphasizing targeted dissection, this approach has the potential to shorten recovery times, reduce morbidity and mortality rates, and improve overall donor comfort. Future research should investigate longer-term outcomes and comparative effectiveness with other surgical modalities to validate and further refine these findings, ultimately guiding optimal practices in living donor nephrectomy.

## Figures and Tables

**Table 1.** Demographics and Preoperative Data

Age, years (range) mean ± SD	(22-50) 38 ± 12
Sex, no (%)	
Male	192 (77%)
Female	58 (23%)
BMI, kg/m^2^ (range) mean ± SD	(23-32) 28.7 ± 2
Relationship, no (%)	
Living related	50 (20%)
Non-related	200 (80%)
Vascular and ureteral variations on preoperative computed tomography, no (%)	
Non-single renal artery	43 (17%)
Non-single renal vein	11 (4.5%)
Ureteral duplication	1 (0.04%)

**Table 2.** Intraoperative Data for the Study Population

Kidney Side, no (%)	
Left	189 (75.6%)
Right	61 (24.4%)
EBL, mL (range) mean ± SD	(60-500)100 ± 40
Operative time, min (range) mean ± SD	(52-170)72.8 ± 16.2
Warm ischemia time, min (range) mean ± SD	(1.5-5) 2.6 ± 1.1
Conversion to open, no (%)/Clavien Grade	3 (1.2%)/IIIb
Need for blood transfusion, no (%)	0 (0%)
Length of incision, cm (range) mean ± SD	(7-12) 9 ± 1.2
Intraoperative complications, no (%)/Clavien grade	
Major vascular injury	2 (0.8%)/IIIb

**Table 3.** Postoperative Findings for the Study Population

Length of hospital stay, days (range) mean ± SD	(1-6) 2.0 ± 1.0
Need for blood transfusion, no (%)/Clavien grade	1 (0.4%)/II
Postoperative complications, no (%)/Clavien Grade	
Wound infection	2 (0.8%)/I
Ileus	4 (1.6%)/II
Incisional hernia	2 (0.8%)/IIIb

**Table 4.** Patient Satisfaction Survey Results

Category	Score (mean ± SD)	Number of Participants
Postoperative pain (VAS)	2.5 ± 1.8	250
Anxiety/Depression (HADS)	6.5 ± 4.3	250
Patient Satisfaction (PSQ)	3.3 ± 1.4	250

Note: The VAS score ranges from 0 (no pain) to 10 (worst pain imaginable). The HADS score ranges from 0 (best) to 21 (worst). The PSQ score ranges from 1 (worst) to 10 (best).

This table summarizes the mean scores for postoperative pain (VAS), anxiety/depression (HADS), and overall patient satisfaction (PSQ).

## Data Availability

The data that support the findings of this study are available on request from the corresponding author.
